# Heat Stress Increases In Vitro Hindgut Fermentation of Distinct Substrates in Iberian Pigs

**DOI:** 10.3390/ani10112173

**Published:** 2020-11-21

**Authors:** Zaira Pardo, Iván Mateos, Rómulo Campos, Andrea Francisco, Manuel Lachica, María José Ranilla, Ignacio Fernández-Fígares

**Affiliations:** 1 Departamento de Fisiología y Bioquímica de la Nutrición Animal, Estación Experimental del Zaidín, CSIC, Profesor Albareda 1, 18008 Granada, Spain; zaira.pardo@eez.csic.es (Z.P.); manuel.lachica@eez.csic.es (M.L.); 2 Departamento de Producción Animal, Universidad de León, Campus de Vegazana s/n, 24071 León, Spain; imata@unileon.es (I.M.); rcamposg@unal.edu.co (R.C.); afranr03@estudiantes.unileon.es (A.F.); mjrang@unileon.es (M.J.R.); 3 Instituto de Ganadería de Montaña, CSIC-Universidad de León, Finca Marzanas s/n, Grulleros, 24346 León, Spain; 4 Departamento de Ciencia Animal, Universidad Nacional de Colombia, Carrera 32 # 12-00, Palmira 76531, Colombia

**Keywords:** heat stress, Iberian pig, in vitro hindgut fermentation, short-chain fatty acids

## Abstract

**Simple Summary:**

Heat stress is a major concern in pig production in summer, as pigs have a limited number of functional sweat glands to transfer body heat. Above 25 °C pigs are out of their comfort zone and mechanisms such as decreasing feed intake or diverting blood from the internal organs to the skin are triggered. Intestinal microbiota is also affected by high ambient temperature but the consequences on fermentation capacity are poorly known. Short-chain fatty acids are the end-products of bacterial metabolism of carbohydrates and protein mainly in the hindgut and, in addition to being a source of energy, they have beneficial effects on immune status and health. An understanding of the effects of heat stress on intestinal fermentation could help to develop strategies mitigating intestinal disorders. We used an in vitro method to assess gas and short-chain fatty acid production, utilizing as inoculum feces from Iberian pigs fed a commercial diet for 28 days under neutral (20 °C) or heat stress (30 °C) conditions. Four substrates with dissimilar fermentation characteristics were incubated in vitro with fecal inoculum for 24 h. Chronic heat stress increased in vitro production of short-chain fatty acids, suggesting a modification of intestinal microbiota activity.

**Abstract:**

Heat stress reduces the feed intake and growth of pigs. We hypothesized that heat stress affects the intestinal fermentation capacity of pigs. Sixteen Iberian pigs (44 ± 1.0 kg) were randomly assigned to one of two treatments (eight pigs/treatment) for 4 weeks—heat stress (HS; 30 °C) ad libitum or thermoneutral (TN; 20 °C) pair feeding. Frozen rectum contents were used as inocula for 24 h in vitro incubations in which a mixture of starches, citrus pectin, inulin from chicory, and cellulose were the substrates. Cellulose was poorly degraded, whereas pectin and the mixture of starches were the most fermentable substrates according to total short-chain fatty acid (SCFA) production. The mixture of starches and inulin produced the greatest amount of gas. For all substrates, heat stress enhanced gas production (8%, *p* = 0.001), total SCFA production (16%, *p* = 0.001), and the production of acetate and propionate (12% and 42%, respectively; *p* = 0.001). The increased isoacid production (33%, *p* = 0.001) and ammonia concentration (12%, *p* = 0.001) may indicate protein fermentation under heat stress. In conclusion, the in vitro intestinal fermentation capacity of pigs under heat stress was increased compared to thermoneutral conditions, which may indicate an adaptive response to heat stress.

## 1. Introduction

The increasing environmental temperature due to global warming affects animal health and production worldwide [1,2]. The main pig producing areas in Spain may be classified as having Mediterranean and hot semi-arid climates (https://en.climate-data.org/europe/spain-5/), where high ambient temperatures are common during summer (July–August), with average highs of 32 °C–36 °C in July. Heat stress reduces feed intake and causes intestinal injury, affecting the growth performance of pigs [3]. Some of these effects are generated by hypoxia caused by redirected blood flow from viscera to the skin [4]. The heat dissipation capacity of pigs is impaired because of their scattered sweat glands, which makes them specifically sensitive to heat stress [5]. The Iberian pig (*Sus mediterraneus*) is the most important Mediterranean native breed, producing cured products of outstandingly high quality. Pure Iberian pigs have traditionally been reared free range, enduring the climatological conditions of each season. Although Iberian pigs are rustic animals, their elevated subcutaneous fat could make them particularly sensitive to the high temperatures of the summer. Heat stress modifies the intestinal microbiota of growing pigs [6], growing-finishing pigs [7] and primiparous sows [8], leading to increased morbidity, as well as altered nutrient digestion and energy metabolism [9]. Microbiota in the large intestine use undigested carbohydrates and protein to substantially generate short-chain fatty acids (SCFA), which are meaningful for the animal as energy and signaling molecules [10]. As in vivo studies are difficult and cumbersome to carry out, in vitro methods are an alternative way to study intestinal fermentation, allowing testing fermentable substrates without the interference of other feed components [11]. The use of in vitro methods to characterize the fermentation of feed ingredients by intestinal microbes and its influence on digestive physiology in pigs is recent [12,13,14,15] compared to the use of these methods in ruminants (e.g., [16,17]). We have used a method based on the in vitro fermentation of a substrate by microbiota from feces of donor pigs, mimicking the fermentation taking place in the large intestine. It is a non-invasive technique for the comparative assessment of fermentation characteristics of feed ingredients and allows for the study of the fermentation capacity of the pig donor. Although the effects of heat stress on SCFA concentrations in different sections of gastrointestinal tracts of pigs have been studied [7,8,18], to our knowledge, there is no information regarding how in vitro fermentation characteristics are affected by heat stress. Furthermore, to the best of our knowledge, the fermentation of pure substrates by Iberian pig inoculum has not been investigated.

We hypothesized that long term heat stress (four weeks, 30 °C) would derange the fermentation capacity of pigs. We chose substrates with distinct fermentation characteristics, as their properties may influence fermentation end-products. The aim of this study was to determine differences in the hindgut fermentation of chemically disparate substrates by Iberian pigs in vitro, as affected by heat stress.

## 2. Materials and Methods

### 2.1. Animals, Treatments, and Diets

Experimental procedures and animal care were in agreement with Spanish Ministry of Agriculture guidelines (RD53/2013). Procedures used were approved by the Bioethical Committee of the Spanish Council for Scientific Research (CSIC, Spain) and the competent local authority (Junta de Andalucía, Spain, project authorization 28/06/2016/118).

Sixteen pure Iberian barrows (44.0 ± 1.00 kg) from Sanchez Romero Carvajal (Puerto de Santa María, Cádiz, Spain) were used. The experimental diet (Table 1) was based on barley-corn-soybean meal, covering all nutrient requirements. The temperature to induce heat stress was chosen according to the average temperature in the hottest months in the main pig producing areas in Spain and was fixed at 30 °C. Average highs above 32 °C–35 °C are not rare in some producing areas in Spain during summer. Accordingly, two experimental treatments were designed (n = 8)—chronic heat stress (HS; 30 °C ± 1 °C, for 4 weeks) fed ad libitum, and thermoneutrality (TN; 22 °C ± 1 °C, for 4 weeks) with pigs pair fed to equal feed intake with HS. For both experimental groups, the temperature was kept constant during the day and night. Feed allowance of pair fed pigs was calculated every day from the estimated daily intake of HS pigs the previous day. The experiment was carried out in 4 replicates, each with two pigs per treatment. Feed intake was calculated on a dry matter basis by collecting feed refusals daily. At arrival, the pigs were randomly assigned to individual partially slatted pens (2 × 1 m) for one week in thermoneutral conditions for acclimatization to facilities. Two separated climate-controlled rooms (TN and HS treatments) were used, in which temperature was controlled using an air conditioning apparatus (LG UM36, LG Electronics Inc., Changwon, South Korea). The temperature and relative humidity inside each room were recorded during the study with the aid of a data logger (HOBO UX100-011; Onset Computer Corporation, Bourne, MA, USA) set to register those values every 15 min. The temperature transition from 22 °C to 30 °C occurred gradually over 8 h at a constant rate of +1 °C per hour.

After the adaptation period, the animals were under TN or HS treatment for four weeks. The pigs in the present experiment were also utilized in a growth trial and were slaughtered at the end of the experimental period (62 ± 1.3 kg) by electrical stunning and instant vertical exsanguination and a sample of rectum content was collected (CO_2_-filled container), rapidly frozen and kept at −20 °C until the fermentation experiment.

### 2.2. Substrates and In Vitro Incubations

Substrates differing in sugar composition and linkages between sugars were selected, as these factors affect fermentation. Starch (glucose polymer with 1,4-alpha linkage) and cellulose (glucose polymer 1,4-beta bond) are homoglucans; inulin is a fructan (fructose or sucrose units linked by β-2,1-linkages); and pectin is a polyuronide (partly branched polymer from d-galacturonic acid with 1,4 linkage). A mix of starches (2 corn starch (SIGMA S-4126), 2 potato starch (SIGMA S-2004), and 1 wheat starch (SIGMA S-2760)); pectin from citrus pulp (SIGMA P-9135); inulin from chicory (SIGMA I-2255-25G); and microcrystalline cellulose (Merck 1-02331.0500) were used as substrates for the in vitro incubations. These substrates were dried overnight at 40 °C and 200 mg of dry substrate were weighted into 120 mL glass bottles.

The day of the incubation, feces were defrosted at room temperature and pooled (an identical amount of feces from two pigs from each experimental condition and an in vivo replicate were used to compose one inoculum). The experiment was repeated in four runs corresponding to the in vivo replicates. In each run, duplicated bottles of each of the five substrates were incubated with both inocula (TN and HS). Blanks (two per inoculum and run) were used to correct the gas production values for gas release from endogenous substrates. Values from the two bottles per substrate and experimental treatments were averaged before statistical analysis, and therefore there were four values per substrate and experimental treatment.

Inoculum preparation was as follows: 25 g of thawed feces and 500 mL of the buffered anaerobic culture medium salts of Goering and Van Soest [19] without trypticase added, were mixed (5% wt/vol final concentration of feces) and homogenized in Stomacher^®^ (model number BA6021, Seward Medical, London, UK) at 230 rpm for one minute. The homogenate was filtered through a nylon bag (200 µm mesh screen). The mixture of diluted feces obtained was added (30 mL) into each bottle under CO_2_ flushing. Bottles were sealed with rubber stoppers and aluminium caps and incubated (39 °C) for 24 h.

### 2.3. Analysis of Samples

After 24 h of incubation, gas production was measured using a pressure transducer (Delta Ohm DTP704-2BGI, Herter Instruments SL, Barcelona, Spain) and a calibrated syringe. A gas sample (10 mL) was stored in an evacuated tube (Terumo Europe N.V., Leuven, Belgium) for the analysis of methane. Bottles were then uncapped and the fermentation was stopped by quenching the bottles in ice water. A liquid sample was immediately obtained (4 mL) from each bottle, mixed with 100 µL of 20% sulfuric acid to preserve the sample and stored at −20 °C until analysis for SCFA and ammonia.

#### 2.3.1. Short-Chain Fatty Acid Analysis

Liquid samples from the bottles were manipulated as described by Saro et al. [20]. Concentrations of SCFA were determined using a GC-2010 gas chromatograph (Shimadzu, Duisburg, Germany). The amounts of SCFA produced were obtained by subtracting the amount present initially in the inoculum from that determined at the end of the incubation period.

#### 2.3.2. Ammonia and Methane Analysis

Liquid samples were defrosted and centrifuged and the supernatant was analyzed for ammonia using colorimetry, following the technique of Weatherburn [21].

Gas samples were analyzed for methane in a gas chromatograph (Shimadzu GC 14B, Shimadzu Europa GmbH, Duisburg, Germany) following the procedures described by Martinez et al. [22].

### 2.4. Statistical Analysis

Data were analyzed using the PROC MIXED of SAS as mixed model, including treatment, substrate and treatment × substrate as fixed effects and incubation run as a random effect. When a significant treatment × substrate (*p* < 0.05) was detected, differences among means were tested using Tukey’s multiple comparison test. Results were considered significant at *p* < 0.05 and trends at 0.05 < *p* < 0.10.

## 3. Results

As anticipated, the feed intake of heat stressed and thermoneutral pair fed pigs used to obtain fecal inocula in the present experiment was similar (2285 g dry matter/day). Additionally, no differences were found in average daily gain (545 g/day), gain:feed (0.24) and final weight (60.3 kg) between TN and HS pigs (unpublished results).

### 3.1. Differences in Substrate In Vitro Fermentation

Gas production and fermentation parameters of the four substrates are shown in Table 2, Table 3 and Table 4. There was a significant substrate × inoculum interaction for most parameters determined (*p* < 0.05), as expected because of the contrasting fermentability characteristics of the substrates.

According to total SCFA (sum of acetate, propionate, butyrate, isoacids, and valerate) and gas production measurements (Table 2 and Table 4), the most fermentable substrates were pectin and the mix of starches, whereas cellulose was hardly fermented. Indeed, the average total SCFA production of pectin was 1900 µmol, compared to 142 µmol for cellulose (Table 2, *p* = 0.001), and the average gas production of the mix of starches was 3209 µmol, compared to 646 µmol for cellulose (Table 4, *p* = 0.001).

Pectin produced the greatest amount of acetate (1523 µmol on average) and cellulose produced the lowest (73 µmol on average) (Table 2). Propionate production was the greatest (*p* = 0.001) for the mix of starches (474 µmol on average), followed by inulin (399 µmol on average), and the lowest was observed for cellulose (29 µmol on average). Butyrate production was the greatest (*p* = 0.001) for the mix of starches (323 µmol on average), followed by pectin and inulin (180 and 148 µmol on average, respectively). Isoacid production was the greatest (*p* = 0.001) for cellulose (14 µmol on average), followed by pectin (10 µmol on average), compared to the rest of substrates (6 µmol on average).

The acetate:propionate ratio (Table 3) was the greatest for pectin compared to the other substrates (12 vs. 3, respectively, *p* = 0.001). The acetate molar proportion was (*p* = 0.001) the highest for pectin (81% on average) and the lowest for cellulose and the mix of starches (52% on average), whereas inulin was intermediate (58% on average). Pectin showed the lowest (*p* = 0.001) propionate molar proportion (9% on average), the mix of starches and inulin showed the highest (27% on average), and cellulose showed an intermediate proportion (18% on average). Pectin showed the lowest butyrate molar proportion (9% on average, *p* = 0.001), whereas the mix of starches was the highest (22% on average). Cellulose had the highest isoacid molar proportion (10% on average, *p* = 0.001) compared to the other substrates (0.4% on average).

Methane production (Table 4) was the lowest (*p* = 0.001) for cellulose (293 µmol on average), with no differences (*p* > 0.10) for the rest of substrates (417 µmol on average).

The ammonia concentration (Table 4) was the largest (*p* = 0.001) for cellulose (241 mg/L on average) and the lowest for the mix of starches (125 mg/L on average), with pectin and inulin showing intermediate values (146 mg/L on average).

### 3.2. Effect of Heat Stress on In Vitro Fermentation

Heat stress increased the capacity of in vitro intestinal fermentation in Iberian pigs, as indicated by augmented total SCFA for all substrates but cellulose (16% across substrates, *p* = 0.001) and gas production (8% across substrates, *p* = 0.001), as shown in Table 2 and Table 4.

Acetate, propionate, and isoacid production were also increased (12%, 42%, and 34%, respectively, across substrates, *p* = 0.001) as a consequence of heat stress (Table 2).

Heat stress increased the propionate molar proportion for pectin and inulin (21% on average, *p* = 0.001) and decreased the acetate:propionate ratio (37%, *p* = 0.001) and the acetate molar proportion only when inulin was the substrate (9%, *p* = 0.001) (Table 3). On the other hand, heat stress increased the ammonia concentration (Table 4) for all substrates (12% across substrates, *p* = 0.001) except for the mix of starches.

No differences (*p* > 0.10) in methane production (Table 4) were caused by heat stress for any incubated substrate.

## 4. Discussion

In vitro gas production techniques used to assess gastrointestinal tract fermentation have limitations but also have many advantages [23]. Although in vitro models usually do not take into account the ongoing production and rapid absorption of SCFAs [24] which occurs in vivo, in vitro fermentation provides a reliable technique to estimate SCFA production, as they are not absorbed [25,26]. In vitro gas production, SCFA production, and ammonia concentration were used as indicators of fermentation in the large intestine [27]. Feces are highly representative of the microbial activity of digesta from the whole large intestine [28] and can be used as a source of inoculum instead of intestinal contents for in vitro fermentation techniques [29,30]. The frozen cecal content and feces of pigs [31,32], horses [33], and rabbits [34] have successfully been used as inoculum to study hindgut fermentation. Finally, we used 24 h incubation time, which is both convenient in the laboratory and close to the estimated transit time of digesta in the large intestine of pigs fed cereal-based diets [35,36].

No information in the literature exists regarding the in vitro fermentation of pure substrates by Iberian pigs. We chose to use a variety of substrates to allow for discrimination in the fermentation capability of inocula, as the extent of fermentation and the profile in SCFAs depend on the substrate [37,38]. The fermentation of dietary fibers is influenced by their chemical characteristics [39,40]. For example, it is known that soluble dietary fibers such as inulin and pectin are generally highly fermentable compared to insoluble fibers [41,42,43], increasing intestinal microbial activity and decreasing transit time [44].

In agreement with previous works using similar substrates [45,46], pectin generated the largest acetate production. The mix of starches showed particularly elevated butyrate production in comparison with the other substrates, in accordance with the literature [46,47]. Butyrate, the fuel for enterocytes [48,49], has beneficial implications for large intestine health [50], as well as for the immune system [51], and may have a trophic effect on the intestinal epithelium [45,52]. The low gas production and total SCFA production reported for cellulose implicate the limited presence of cellulolytic microbiota, which is supported by the elevated ammonia concentration, similar to the blank (data not shown), indicating that ammonia was not used by microbiota. On the other hand, the reduced concentration of ammonia and production of isoacids by inulin and the mix of starches indicates reduced protein fermentation, which is in agreement with the reduced protein fermentative end-products and bacterial populations associated when fructans were the substrate of fermentation in vivo in pigs, humans, and dogs [53,54,55]. The fermentation of protein generates ammonia and amines, which are considered toxic for the animal [56,57].

Even when direct comparison between results from different in vitro fermentation studies is not possible, the extent of degradation of the substrates used in the present experiment is in line with those found by other authors when using pectins and starch of different origins [12], hydrolyzed sugar beet pulp [58], or soy pectin and oligofructose [59]. The elevated proportion of acetate after in vitro fermentation of all substrates assayed is in accordance with the literature [60,61,62]. Likewise, cellulose was poorly used by bacteria, concurring with most fermentation or digestibility studies [63,64,65]. Other studies, however, have shown that a longer incubation time (72 h) is necessary for cellulose to reach a total gas production comparable to soluble fibers [66,67].

The thermoneutral zone of pigs is between 18 °C and 25 °C and temperatures above 25 °C activate thermoregulatory responses [68]. We chose 22 °C as thermoneutral and 30 °C as heat stress temperatures to study the effect of chronic heat stress on the fermentation capacity of pigs in vitro. Additionally, to study the direct effects of heat stress independent of feed intake (heat stress decreases feed intake), pigs in thermoneutral conditions were feed-restricted to assure the same level of intake.

We have found no information on the possible effects of heat stress on the fermentation capacity of pigs in vitro—only limited information about volatile fatty acid concentration in feces or in the hindgut is available [7,8,18].

The acetate:propionate ratio is used in ruminants to characterize the kind of predominant fermentation in the rumen. The lower the ratio, the more efficient. Since propionate is glucogenic, a lower acetate:propionate ratio, as in Iberian HS pigs in the present experiment, indicates increased production and availability of energy. Unexpectedly, heat stress increased total SCFA content for all substrates utilized in our study. Experiments with modern breeds have shown decreased concentrations of SCFA in the feces of growing Duroc × Large White × Landrace pigs (30 kg) subjected to acute heat stress (35 °C for 24 h) [7] and in the feces of late gestational Landrace × Large White primiparous sows exposed to chronic heat stress (28 °C–32 °C for 22 days) [8]. Interestingly, the SCFA content in the cecum of finishing pigs fed ad libitum and subjected to daily cyclical heat stress (37 °C for 9 h and 27 °C for 15 h) for 28 days was not altered [18]. Temperature and duration of experiments, as well as feed intake, seem to be among the key factors affecting intestinal microbiota and may certainly be responsible for discrepancies between studies. The breed may also play an important role regarding microbiota composition. For instance, gut microbiota is a major contributor to adiposity in pigs [69]. Augmented SCFA may be considered advantageous for the pig as SCFAs promote resistance to opportunistic pathogens including enterotoxigenic *Escherichia coli*, *Clostridum*, and *Salmonella* [54,70]. Additionally, microbial degradation of fiber to SCFA might contribute to the energy maintenance requirements of the pig to a considerable extent (15%–30%) [71,72]. Finally, butyrate plays a main role as the preferential fuel of enterocytes [48,49] and has been linked to improved gut health [73].

It has recently been reported that fecal microbiota composition is significantly influenced by climatic conditions in growing pigs [6], so it is possible that in the present study, the microbiota of the pigs adapted to heat stress conditions after 4 weeks. Iberian pigs are rustic animals [74] that may be resilient to different stresses. Our results suggest such a resiliency, as the pigs showed increased microbial activity in the large intestine under chronic heat stress. Further research is required to provide evidence on the effect of heat stress on microbiota composition and function in the hindgut of Iberian pigs.

## 5. Conclusions

Heat stress increased the in vitro hindgut fermentation capacity in Iberian pigs. If confirmed in vivo, the augmented SCFA production could be considered a resilience mechanism that limits the negative effects of heat stress.

## Figures and Tables

**Table 1:** Composition and chemical analysis (g/kg) of the experimental diet.

**Ingredients**	
Barley grain	700
Corn	143.7
Soybean meal	127
Calcium phosphate	9.3
Calcium carbonate	6.2
Sodium chloride	3.0
Vitamins and minerals	3.0
L-Lysine (50%)	5.0
L-Threonine (50%)	2.1
Methionine hydroxy-analog (75%)	0.7
**Chemical Analysis**	
Dry matter	899
Ash	49.0
Crude protein	141.1
Crude fiber	41.0
Ether extract	19.0
Calcium	6.8
Phosphorous	6.3
Sodium	1.6
Lysine	9.0
Methionine	2.4
Gross energy (MJ/kg)	16.6

**Table 2:** Individual and total short-chain fatty acid (SCFA, µmol) production after 24 h in vitro fermentation of four substrates (Subs) by fecal inocula obtained from pigs under thermoneutral (TN) or heat stress (HS) conditions.

		Treatment			*p*-Value		
SCFA Production	Subs	TN	HS	SEM ^1^	Subs	Trt	Subs × Trt
Total SCFA	Mix of starches	1546 ^A^	1809 ^A^*	15.4	0.001	0.001	0.001
	Pectin	1789 ^B^	2011 ^B^*				
	Inulin	1211 ^C^	1410 ^C^*				
	Cellulose	118 ^D^	166 ^D^				
Acetate	Mix of starches	788 ^A^	947 ^A^*	12.2	0.001	0.001	0.001
	Pectin	1442 ^B^	1603 ^B^*				
	Inulin	741 ^A^	765 ^C^				
	Cellulose	62.0 ^C^	84.0 ^D^				
Propionate	Mix of starches	438 ^A^	509 ^A^*	7.9	0.001	0.001	0.001
	Pectin	151 ^B^	221 ^B^*				
	Inulin	285 ^C^	512 ^A^*				
	Cellulose	23.0 ^D^	35.0 ^C^				
Butyrate	Mix of starches	303 ^A^	343 ^A^*	4.4	0.001	0.366	0.001
	Pectin	185 ^B^	174 ^B^				
	Inulin	177 ^B^	119 ^C^*				
	Cellulose	20.0 ^C^	28.0 ^D^*				
Isoacids ^2^	Mix of starches	6.1 ^A^	8.6 ^AB^	0.37	0.001	0.015	0.434
	Pectin	8.1 ^B^	11.4 ^BC^				
	Inulin	4.2 ^A^	4.7 ^A^				
	Cellulose	11.6 ^C^	15.4 ^C^*				
Valerate	Mix of starches	2.2 ^A^	1.9 ^A^	0.14	0.039	0.001	0.001
	Pectin	3.6 ^B^	1.5 ^A^*				
	Inulin	3.3 ^B^	1.2 ^B^*				
	Cellulose	1.8 ^A^	3.6 ^C^*				

^1^ Standard error of mean. ^2^ Isoacids: isobutyrate + isovalerate. ^A,B,C,D^ Means within a column with different superscript are significantly different; *p* < 0.05. * Within a row, means with * are significantly different; *p* < 0.05.

**Table 3:** Acetate:propionate ratio and molar proportions of short-chain fatty acids after 24 h in vitro fermentation of four substrates (Subs) by fecal inocula obtained from pigs under thermoneutral (TN) or heat stress (HS) conditions.

		Treatment			*p*-Value		
Variable	Subs	TN	HS	SEM ^1^	Subs	Trt	Subs × Trt
Acetate/Propionate	Mix of starches	3.6 ^A^	2.4 ^A^*	0.25	0.001	0.001	0.001
	Pectin	15.6 ^B^	9.2 ^B^*				
	Inulin	3.1 ^A^	2.1 ^A^*				
	Cellulose	3.9 ^A^	3.5 ^C^				
Molar proportions							
Acetate	Mix of starches	51.0 ^A^	52.2 ^AB^	0.51	0.001	0.097	0.029
	Pectin	81.0 ^B^	80.2 ^C^				
	Inulin	61.0 ^C^	55.7 ^A^				
	Cellulose	54.3 ^D^	50.5 ^B^				
Propionate	Mix of starches	25.2 ^A^	26.4 ^A^	0.35	0.001	0.001	0.001
	Pectin	8.1 ^B^	10.7 ^B^*				
	Inulin	23.3 ^A^	33.8 ^C^*				
	Cellulose	17.0 ^C^	18.0 ^D^				
Butyrate	Mix of starches	22.4 ^A^	20.8 ^A^	0.38	0.001	0.012	0.030
	Pectin	10.2 ^B^	8.5 ^B^*				
	Inulin	14.9 ^C^	9.6 ^B^*				
	Cellulose	17.2 ^D^	16.8 ^C^				
Isoacids ^2^	Mix of starches	0.41 ^A^	0.48 ^A^	0.15	0.001	0.021	0.004
	Pectin	0.45 ^A^	0.56 ^A^				
	Inulin	0.39 ^A^	0.31 ^A^				
	Cellulose	10.1 ^B^	10.4 ^B^				
Valerate	Mix of starches	0.14 ^A^	0.10 ^A^*	0.06	0.001	0.365	0.009
	Pectin	0.19 ^A^	0.08 ^A^*				
	Inulin	0.24 ^A^	0.60 ^B^*				
	Cellulose	1.16 ^B^	1.02 ^C^				

^1^ Standard error of mean. ^2^ Isoacids: isobutyrate + isovalerate. ^A,B,C,D^ Means within a column with different superscript are significantly different; *p* < 0.05. * Within a row, means with * are significantly different; *p* < 0.05.

**Table 4:** Gas and methane (CH_4_) production (µmol) and concentrations of ammonia (mg/L) after 24 h in vitro fermentation of four substrates (Subs) by fecal inocula obtained from pigs under thermoneutral (TN) or heat stress (HS) conditions.

		Treatment			*p*-Value		
Variable	Subs	TN	HS	SEM ^1^	Subs	Trt	Subs × Trt
Gas production	Mix of starches	3079 ^A^	3338 ^A^*	13.5	0.001	0.001	0.002
	Pectin	3000 ^AB^	3296 ^A^*				
	Inulin	2912 ^B^	3001 ^B^*				
	Cellulose	584 ^C^	707 ^C^*				
CH_4_ production	Mix of starches	402 ^A^	412 ^A^	5.5	0.001	0.319	0.045
	Pectin	426 ^A^	457 ^A^				
	Inulin	402 ^A^	404 ^A^				
	Cellulose	293 ^B^	236 ^B^*				
Ammonia	Mix of starches	121 ^A^	128 ^A^	1.1	0.001	0.001	0.040
	Pectin	145 ^B^	171 ^B^*				
	Inulin	124 ^A^	144 ^C^*				
	Cellulose	230 ^C^	252 ^D^*				

^1^ Standard error of mean. ^A,B,C,D^ Means within a column with different superscript are significantly different; *p* < 0.05. * Within a row, means with * are significantly different; *p* < 0.05.
